# Interpreting results of cluster surveys in emergency settings: is the LQAS test the best option?

**DOI:** 10.1186/1742-7622-5-25

**Published:** 2008-12-09

**Authors:** Oleg O Bilukha, Curtis Blanton

**Affiliations:** 1 Division of Emergency and Environmental Health Services, National Center for Environmental Health, Centers for Disease Control and Prevention, Atlanta, Georgia 30341, USA

## Abstract

Cluster surveys are commonly used in humanitarian emergencies to measure health and nutrition indicators. Deitchler *et al*. have proposed to use Lot Quality Assurance Sampling (LQAS) hypothesis testing in cluster surveys to classify the prevalence of global acute malnutrition as exceeding or not exceeding the pre-established thresholds. Field practitioners and decision-makers must clearly understand the meaning and implications of using this test in interpreting survey results to make programmatic decisions. We demonstrate that the LQAS test–as proposed by Deitchler *et al*. – is prone to producing false-positive results and thus is likely to suggest interventions in situations where interventions may not be needed. As an alternative, to provide more useful information for decision-making, we suggest reporting the probability of an indicator's exceeding the threshold as a direct measure of "risk". Such probability can be easily determined in field settings by using a simple spreadsheet calculator. The "risk" of exceeding the threshold can then be considered in the context of other aggravating and protective factors to make informed programmatic decisions.

## Introduction

Cluster surveys are often used in humanitarian emergencies to measure important nutrition and health indicators. A majority of such surveys measure the prevalence of global acute malnutrition (GAM) in children aged 6 to 59 months, a key nutritional indicator used to define the presence and gravity of an emergency. Important decisions about implementing large-scale interventions, such as general food distribution and/or feeding programs, are often based principally or in part on the prevalence of GAM. Several international organizations have published guidelines for implementing food and nutrition interventions in emergencies, where the need for such interventions is determined on the basis of the prevalence of GAM relative to pre-determined thresholds (5%, 10%, 15%) and the presence of aggravating factors, such as high mortality rates, epidemics of certain infectious diseases, or poor food security [1,2].

A conventional way of analyzing GAM in a population is to estimate the prevalence from a cluster survey and then compute a 95% confidence interval around the estimate [3,4]. Deitchler and colleagues [5,6] recently proposed using decision rules based on the lot quality assurance (LQAS) method to classify the prevalence of GAM in cluster emergency nutrition surveys vis-à-vis pre-established thresholds. The authors also proposed using cluster designs with a reduced number of individuals per cluster and a reduced overall sample size of about 200, compared with 900 individuals in a "conventional" 30 × 30 (30 clusters by 30 children) design. The implications of these proposed designs on precision, validity and resources required to complete the survey have been discussed in detail in a recent paper [7].

Since the LQAS method has not been routinely used to analyze nutrition cluster survey data, we consider it important to provide a simple explanation to field practitioners of how this test is conducted, what it means, and why there may be apparent discrepancies between the results of the LQAS decision rule method and the observed prevalence of GAM. It is important also to consider how this proposed method for decision-making compares to existing practices and to explore the issue of whether there are better statistical options available to compare survey prevalence estimates to preset thresholds.

## Discussion

The LQAS hypothesis test used by Deitchler and colleagues is formulated as:

H_o_: *p *≥ *p*_0 _vs. H_a_: *p *<*p*_0_

where *p *is the true population prevalence and *p*_0 _is the threshold of interest to which the actual prevalence is compared [5,6].

In other words, it is a one-sided test to determine, at a given level of confidence α (in Deitchler *et al*., α is set to 0.1), whether the true population value is *lower *than some threshold value *p*_0_. Unfortunately, this test as proposed by Deitchler *et al*. provides no information concerning the probability of the true value being *higher *than the threshold. This can be illustrated with a simple example.

The LQAS hypothesis test is performed by counting the number of GAM cases in the survey sample and comparing this count to a pre-established decision rule number [8]. For example, Deitchler and colleagues, using α = 0.1, classify the true population value of GAM as <10% if the count of GAM cases in a 33 × 6 (33 clusters of 6 children) or a 67 × 3 (67 clusters of 3 children) cluster survey is 13 or less, and they declare the population value of GAM to be ≥ 10% if the count of GAM cases in such survey is 14 or more [5]. The decision rule numbers are derived from analyzing binomial probability distributions, as described in detail elsewhere [9]. The sample sizes in 33 × 6 and 67 × 3 surveys proposed by Deitchler and colleagues are close to 200 (198 in 33 × 6 and 201 in 67 × 3); therefore, the threshold prevalence of 10% corresponds to 20 GAM cases, and the decision rule number of 14 GAM cases (when according to LQAS rule the prevalence is classified to be ≥ 10%) corresponds to a prevalence of 7%.

In general, at each prevalence threshold, one can formulate a one-sided LQAS hypothesis test in two ways:

1. The null hypothesis is that the true value is *greater than or equal *to the threshold, and the alternative is that the true value is *below *the threshold (as formulated by Deitchler and colleagues). In this case, to reject the null, the observed prevalence should be somewhat lower than the threshold (for example, for the threshold of 10% the authors specify the cutoff value of 13, which corresponds to the prevalence of about 6.5%). In this case, if the observed count of cases is 13 or below, the null should be rejected and the alternative hypothesis accepted that the true prevalence is below 10%. If however, the count of cases is 14 or above, it would be incorrect to declare that the true prevalence is 10% or above but only that the null hypothesis cannot be rejected. This is a fundamental difference, as explained below.

2. The null hypothesis is that the true value is *less than or equal to *the threshold, and an alternative that the true value is *above *the threshold. In this case, to reject the null, the observed prevalence should be somewhat higher than the threshold. Using α = 0.1, if the observed count of cases is 26 or above (which corresponds to a prevalence of 13%), the null hypothesis is rejected and the alternative accepted that the true prevalence is above 10%. If, however, the count of cases is 25 or below, it would be incorrect to conclude that the true prevalence is 10% or lower, only that we cannot reject the null hypothesis.

Therefore, for counts of 13 and below, it can be declared (at alpha = 0.1) that the true population prevalence is below 10% and for counts of 26 or above the true prevalence is above 10%. For the counts in the "gray area" of 14 to 25 that correspond to prevalences from about 7% to 13%, neither the former nor the latter statement can be made because we can neither reject the null of 10 or below nor the null of 10 and above. Using the first one-sided test as a screening tool for "high risk" (i.e., exceeding the 10% threshold) areas would result in high sensitivity but low specificity (i.e., producing few false-negative but many false-positive results), whereas using the second one-sided test would result in low sensitivity and high specificity (i.e., producing few false-positives but many false-negatives).

As can be inferred from the above example, applying a one-sided LQAS test as proposed by Deitchler and colleagues would identify areas where the true population prevalence is below the threshold with few false negatives (i.e., few areas where GAM is in reality above the threshold will be declared as being below the threshold). It will, however, have a substantial propensity to produce false-positives (i.e., declare areas with true GAM prevalence below the threshold to be above the threshold). This is because areas with measured GAM prevalence as low as 7% (corresponding to 14 GAM cases) would be declared to be above the 10% threshold, while areas with measured GAM prevalence as low as 12% would be declared to be above the 15% threshold. Table 1 shows the probability of the true population value's exceeding the 10% GAM threshold for GAM counts that exceed the LQAS decision rule for a 10% threshold. This probability is the p-value of the one-sided t-test that the true population prevalence is lower than the threshold. As can be seen, the LQAS decision rules would declare the GAM prevalence as exceeding the threshold in situations where the statistical probabilities of exceeding the threshold are as low as 10%–15%.

**Table 1:** The probability* of the true population value of GAM exceeding the 10% threshold for different counts of GAM cases and different design effects in a 33 × 6 (33 clusters of 6 children) survey.

Observed GAM count	GAM prevalence	Probability of GAM exceeding 10% threshold, %			
		
		DEFF** = 1	DEFF** = 1.1	DEFF** = 1.2	DEFF** = 1.3
14	7.1%	9.0%	10.0%	10.9%	11.9%

15	7.6%	13.2%	14.3%	15.4%	16.3%

16	8.1%	18.7%	19.9%	20.9%	21.8%

17	8.6%	25.6%	26.6%	27.5%	28.2%

18	9.1%	33.6%	34.4%	35.0%	35.5%

19	9.6%	42.5%	42.9%	43.2%	43.5%

20	10.1%	51.9%	51.8%	51.7%	51.6%

21	10.6%	61.0%	60.6%	60.2%	59.8%

22	11.1%	69.6%	68.9%	68.1%	67.5%

23	11.6%	77.1%	76.2%	75.3%	74.5%

* Based on the one-sided t-test with 32 degrees of freedom

** Design effect

Table 2 provides another illustration of the propensity of the LQAS test proposed by Deitchler and colleagues to produce false positive classifications. This table is based on the operating characteristic curve for the LQAS binomial test, assuming a survey sample size of 198 and a decision rule of 13 GAM cases to reject the null hypothesis. It presents the probability of the LQAS test failing to reject the H_0_: *p *≥ 10% at different levels of true population prevalence of GAM. As can be seen, in areas where the true population prevalence of GAM is 6%, the LQAS test has a 30% probability of classifying the area as ≥ 10% GAM. In other terms, if 100 surveys are conducted is areas where the true population prevalence of GAM is 6%, on average 30 of these areas will be classified by the LQAS test as having GAM ≥ 10%. This probability of "false positive" classification increases to 52% for areas with true GAM prevalence of 7%, and to 72% for areas with true GAM prevalence of 8%.

**Table 2:** The probability* (y) of the LQAS test failing to reject the null hypothesis H_0_: *p *≥ 10% for different levels of the true population prevalence (x).

True population prevalence of GAM	5%	6%	7%	8%	9%
Probability of failing to reject H_0_: *p *≥ 10%	12.3%	30.2%	52.4%	72.2%	86.0%

* Based on binomial test

Assuming survey sample size of 198 and decision rule (count of GAM cases to reject null) of 13 (n = 198, d = 13).

This approach of identifying all areas that may potentially be at risk, without much concern for specificity, may be justified in other situations where LQAS is applied. However, it may not be suitable for making decisions about GAM prevalence in humanitarian emergencies, where erroneous multi-million dollar funding decisions may be made or uncalled-for interventions implemented on a mass scale, thus diverting scarce resources from other life-saving programs, potentially putting program staff at unnecessary risk, or undermining local food production by unjustifiably flooding local markets with food aid. It is also not immediately obvious whether this approach adds any value to the conventional method of estimating the prevalence, which involves constructing a 95% confidence interval and considering it vis-à-vis the threshold of interest to make programmatic decisions.

Important questions about the appropriateness of the currently used GAM thresholds (5%, 10%, 15%), their evidence base, and whether the concept of making decisions based on comparing the observed GAM prevalence to thresholds is meaningful or appropriate in all humanitarian situations is a subject of a separate debate and is beyond the scope of this paper. It seems, however, that currently the most common way of classifying GAM relative to the thresholds is largely based on the observed prevalence estimate (e.g., if the GAM prevalence observed in the survey exceeds the threshold, then the area is declared above the threshold, and vice-versa). From a purely statistical perspective, this means that GAM is declared above the threshold when the statistical probability of the true population value of GAM exceeding the threshold is above 50%. This method, theoretically, would produce as many false-positive as false-negative results. One drawback of this approach is that the width of the confidence interval becomes virtually irrelevant; it may be, in fact, often ignored in summarizing the data for decision-making. On the other hand, in the LQAS decision-making algorithm advocated by Deitchler and colleagues, GAM is classified as being above the threshold when the statistical probability of the true population value of GAM exceeding the threshold is 10% or higher. It is, therefore, a quite conservative approach compared to the existing practice and prone to producing many false-positives and few false-negatives, as illustrated above.

One clear similarity between these two approaches, however, is that they both reduce rich statistical information to a simple yes/no answer–one at a 50% probability level, the other at 10% probability. We do not intend to discuss which of these two is preferable, or what level of probability (10%, 30%, 50% or other) decision-makers should use. We would rather argue that whenever the decision-making process involves comparing observed values to thresholds, it would make sense to report the *statistical probability *of the true population value's exceeding the threshold in addition to the point estimate and 95% confidence interval. This probability provides a direct measure or "risk" that GAM in this population is higher than the threshold, and it can then be considered in the context of other existing and potential risk factors to make informed programmatic decisions. Such statistical probability is easily calculated from survey data, and it can be made available to field practitioners using a simple Excel-based calculator, where users enter the number of clusters, total sample size, observed design effect, and the number of GAM cases (or GAM prevalence) in the survey sample. This simple calculator is available on request from the authors of this paper.

For example (from Table 1), if the count of GAM cases in a 33 × 6 survey is 17 and the design effect is 1.2, the estimated probability of the true population value of GAM exceeding the 10% threshold is 27.5%. Decision-makers could then use the 27.5% "risk" along with other risk factors to make an appropriate (and informed) programmatic decision.

## Conclusion

In conclusion, it is critical that field practitioners and decision-makers clearly understand the meaning and implications of using the LQAS test to interpret cluster survey results as proposed by Deitchler and colleagues. As discussed, this test has a potential to produce false-positive results that suggest interventions in situations where interventions may not be needed. If it is critical for decision-making to compare the observed prevalence of an indicator like GAM with the pre-set threshold, we suggest as an alternative reporting the probability of the true population prevalence's exceeding the threshold as a direct measure of "risk." This "risk" can then be considered in the context of other aggravating and protective factors to make informed programmatic decisions.

While, as discussed in this paper, the LQAS hypothesis test may not be an optimal option for interpreting the results of nutrition cluster surveys in emergencies, it remains a valuable technique of choice for many other public health applications, especially where quick and inexpensive screening method for a single indicator of interest is needed [10].

## Competing interests

The authors declare that they have no competing interests.

## Authors' contributions

OOB designed the study, OOB and CB performed analysis, wrote and revised the manuscript.
